# Online hyphenation of centrifugal partition chromatography with countercurrent chromatography (CPC-CCC) and its application to the separation of saturated alkylresorcinols

**DOI:** 10.1007/s00216-022-04136-x

**Published:** 2022-05-31

**Authors:** Tim Hammerschick, Walter Vetter

**Affiliations:** Institute of Food Chemistry, Department of Food Chemistry (170b), University of Hohenheim, 70599 Stuttgart, Germany

**Keywords:** Alkylresorcinol, Centrifugal partition chromatography, Countercurrent chromatography, Countercurrent separation, Liquid–liquid chromatography

## Abstract

Centrifugal partition chromatography (CPC) and countercurrent chromatography (CCC) are two preparative techniques mainly used for the isolation and purification of natural products. While CPC benefits from a larger sample capacity, CCC typically provides better peak resolutions and hereby higher purities. In this study, we aimed to combine both advantages by the direct linking of CPC and CCC which was achieved by installation of switching valves and connection tube. The hyphenated CPC-CCC setup was tested with major alkylresorcinols which were obtained from a transesterified and hydrogenated rye extract. Injections of 1- and 5-g samples into the individual CCC system confirmed the limited sample capacity because of immediate flooding with the 5-g sample (total loss of stationary phase). In comparison, the CPC system was stable with 5- and 10-g samples but the peak resolution with 1-g sample was poorer than with the CCC system. Injections of 5- and 10-g samples into the CPC-CCC system were successful. However, a sample load of 10 g resulted in lower purities of the alkylresorcinols (80% or less) due to peak tailing. By contrast, injection of 5-g sample provided high amounts of ~ 1.2 g alkylresorcinols with purities of > 95%.

**Graphical abstract:**

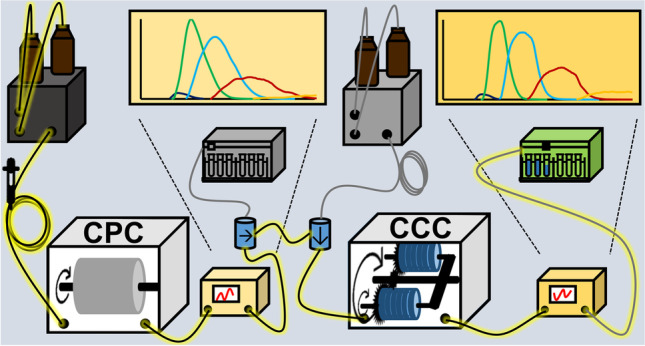

**Supplementary Information:**

The online version contains supplementary material available at 10.1007/s00216-022-04136-x.

## Introduction

Countercurrent separation (CCS) is a summarizing term for support free, all-liquid chromatographic techniques widely used in the preparative isolation and purification of natural and synthetic products [1–4]. CCS benefits from a high loading capacity, freely selectable stationary and mobile phases, low solvent consumption, and a low risk of sample denaturation [5–8]. CCS can be subdivided into hydrostatic centrifugal partition chromatography (CPC) and hydrodynamic countercurrent chromatography (CCC). In CPC, separations take place in disks or cartridges with engraved chambers or cells interconnected by ducts which are arranged around a central axis of rotation [9, 10]. Due to the constant centrifugal field, the stationary phase is maintained in the separation chambers/cells while the pumped mobile phase is percolating through it [11]. In contrast, CCC separations take place in a hollow tube wound around a bobbin, which rotates in a planetary motion about both itself and a central axis [12]. This movement generates a variable gravity field and a force (Archimedean screw effect), which is the retention mechanism of the stationary phase and provides a mechanism for alternating mixing and settling zones inside the coil [1]. Major differences between CCC and CPC can be summarized by the following rule of thumb. Compared to CCC, CPC offers more control over the stationary phase due to the constant gravity field (hydrostatic system), which prevents flooding of the stationary phase even at higher flow rates [8, 9]. In addition, CPC is particularly suitable for biphasic solvent systems with a low difference in density, which is typical of very hydrophilic or aqueous biphasic solvent systems [7, 13]. Moreover, the higher stability of the solvent system is linked with a higher sample capacity in CPC [2, 14]. Vice versa, CCC benefits from a higher partition efficiency which results in sharper peaks compared to CPC [6, 8, 9, 15, 16]. However, the better chromatographic resolution of CCC systems is paired with lower sample loads. Injections of too large amounts are accompanied with severe bleed or even total loss of the stationary phase [17]. For example, the CCC system used in our laboratory (2.1-mm i.d. coils) tolerates maximum sample loads of only ~ 1 g for lipids [18, 19].

In this study, we aimed to overcome this restriction in the sample load of CCC by online linking of CPC and CCC instruments. The rationale behind this concept was that CPC would serve as an injection and pre-separation tool. Namely, the initial CPC separation will distribute the sample initially injected so that smaller and manageable amounts of analytes will enter the CCC system at any given moment. Hence, higher sample amounts can be injected relative to conventional CCC runs. Here, we describe the setup of the direct coupling of CPC with CCC and test it with focus on the separation and isolation of major saturated, odd-chained alkylresorcinols (5-alkyl-1,3-dihydroxybenzenes, ARs). ARs are a class of bioactive amphiphilic lipid compounds which are mainly found in cereals [20–22]. In particular, rye grains have a high AR content (~ 0.5–1.0 mg/g) with predominance of saturated, odd-chained compounds (AR15:0–AR25:0, Fig. 1) [23–25]. Prior to CCS, ARs were extracted from rye grains followed by transesterification of interfering fatty acids and hydrogenation of unsaturated ARs. In the following, the CCS fractionation of individual saturated, odd-chained ARs was evaluated. Different sample amounts (5 g and 10 g) were injected into the CPC-CCC system and the results were compared with conventional CCC (1- and 5-g samples) and CPC (1-, 5-, and 10-g samples) runs.

**Fig. 1:**
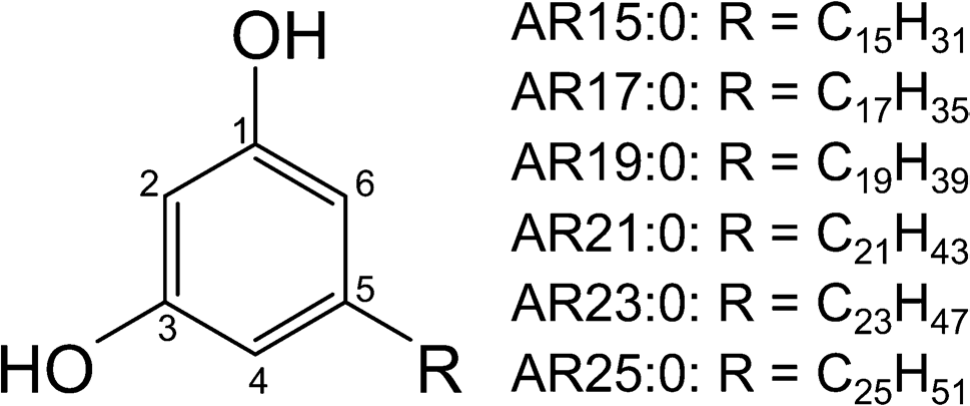
General chemical structures of major alkylresorcinols in rye evaluated in this study: 5-*n*-pentadecyl-1,3-dihydroxybenzene (AR15:0); 5-*n*-heptadecyl-1,3-dihydroxybenzene (AR17:0); 5-*n*-nonadecyl-1,3-dihydroxybenzene (AR19:0); 5-*n*-heneicosyl-1,3-dihydroxybenzene (AR21:0); 5-*n*-tricosyl-1,3-dihydroxybenzene (AR23:0); and 5-*n*-pentacosyl-1,3-dihydroxybenzene (AR25:0)

## Materials and methods

### Chemicals, standard, and rye sample

Pyridine (> 99%, distilled before use), ethyl acetate (> 99%), and platinum(IV) oxide (PtO_2_) were purchased from Sigma-Aldrich (Steinheim, Germany), whereas cyclohexane (> 99%), methanol, and *n*-hexane (both HPLC grade) were from Th. Geyer (Renningen, Germany). Cyclohexane and ethyl acetate were combined (1:1, *v/v*) and distilled to give the azeotropic mixture CE_az_ (46:54, *w/w*). Tetrahydrofuran (THF) (> 99%) (distilled and stored over a 3-Å molecular filter before use), sulfuric acid (96%), anhydrous sodium sulfate, and sodium chloride (> 99.5%) were from Carl Roth (Karlsruhe, Germany). Silylation agent N,O-bis(trimethylsilyl)trifluoroacetamide (BSTFA) was from Macherey–Nagel (Düren, Germany) and helium, hydrogen (both 5.0 quality), and nitrogen (99.95%) were from Westfalen company (Münster, Germany). Demineralized water was prepared in-house. An internal standard solution of docosanoic acid methyl ester (22:0-ME) in *n*-hexane with a concentration of 5 μg/mL was prepared after docosanoic acid was transesterified with 1% sulfuric acid in methanol according to Wendlinger and Vetter [26]. Whole rye grains were obtained in a local supermarket in Stuttgart (Germany).

### Sample preparation

**Fig. 2:**
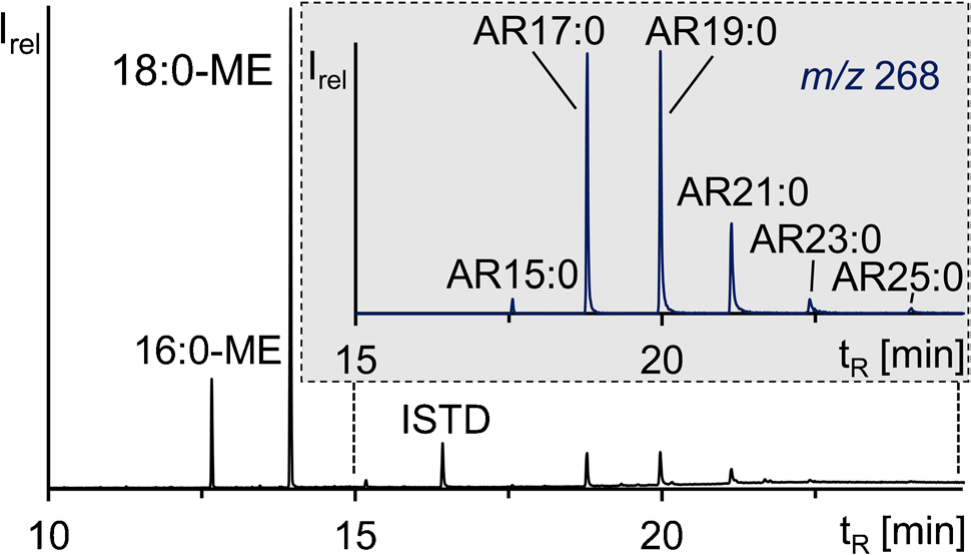
GC/MS chromatogram (full scan) of an aliquot of the combined transesterified and hydrogenated rye grain extracts after silylation with an enlarged insert of the ion trace *m*/*z* 268 from the full scan

Hydrogenated, transesterified rye extract was prepared according to Hammerschick et al. [27]. In brief, three batches of 3-kg whole rye grains, cold extracted using 2.2 L CE_az_, provided ~ 15.8 g (0.53%), ~ 14.4 g (0.48%), ~ 14.7 g (0.49%) rye grain extracts (Table 1) which were transesterified (4 h, 80 °C under reflux) with 300 mL 1% sulfuric acid in methanol, respectively. The final *n*-hexane extracts (~ 12.7 g, ~ 11.7 g, ~ 11.9 g, Table 1) were hydrogenated (15 mL THF, 3 mg PtO_2_ catalyst, H_2_ atmosphere, 40 °C for 4 days), weighed (same weights as after the previous step), and pooled. An aliquot of the transesterified and hydrogenated extract was silylated (10**–**80-μg sample, 25 μL pyridine, 50 μL BSTFA, 30 min at 60 °C [23]) and analyzed by gas chromatography with mass spectrometry (GC/MS) (FAME pattern: ~ 85% 18:0-ME, 14% 16:0-ME, 1% 20:0-ME; AR pattern: 1% AR15:0, 34% AR17:0, 38% AR19:0, 19% AR21:0, 5% AR23:0, and 2% AR25:0 (Fig. 2)). Shares of this pooled extract were separated in different amounts by CCS.

**Table 1:** Masses of the individual preparation steps (extraction, esterification, and hydrogenation) before isolation of the saturated ARs by CCS

Pre-treatment	Batch 1	Batch 2	Batch 3
Mass rye grains	3000 g	3000 g	3000 g
Mass extract	~ 15.8 g (0.53%)	~ 14.4 g (0.48%)	~ 14.7 g (0.49%)
Mass for esterification	~ 15.8 g	~ 14.4 g	~ 14.7 g
Mass after esterification	~ 12.7 g (80.4%)	~ 11.7 g (81.1%)	~ 11.9 g (81.0%)
Mass for hydrogenation	~ 12.7 g	~ 11.7 g	~ 11.9 g
Mass after hydrogenation	~ 12.7 g	~ 11.7 g	~ 11.9 g
Total mass of sample for CCS	36.3 g

### Countercurrent separations

#### Solvent system and elution mode

CPC, CCC, and CPC-CCC separations were performed with the solvent system HEMWat − 7 (*n*-hexane–ethyl acetate–methanol–water (9:1:9:1, *v/v/v/v*)) [27, 28]. For each CCS run, a new batch of the solvent system was prepared by combining 900 mL *n*-hexane, 100 mL ethyl acetate, 900 mL methanol, and 100 mL water. The resulting mixture was vigorously shaken several times in a separating funnel. After 30 min of equilibration, the phases were separated and then degassed for 15 min. The lower (more polar) phase was used as the mobile phase (named descending mode in CPC and head-to-tail mode in CCC).

#### CPC system

**Fig. 3:**
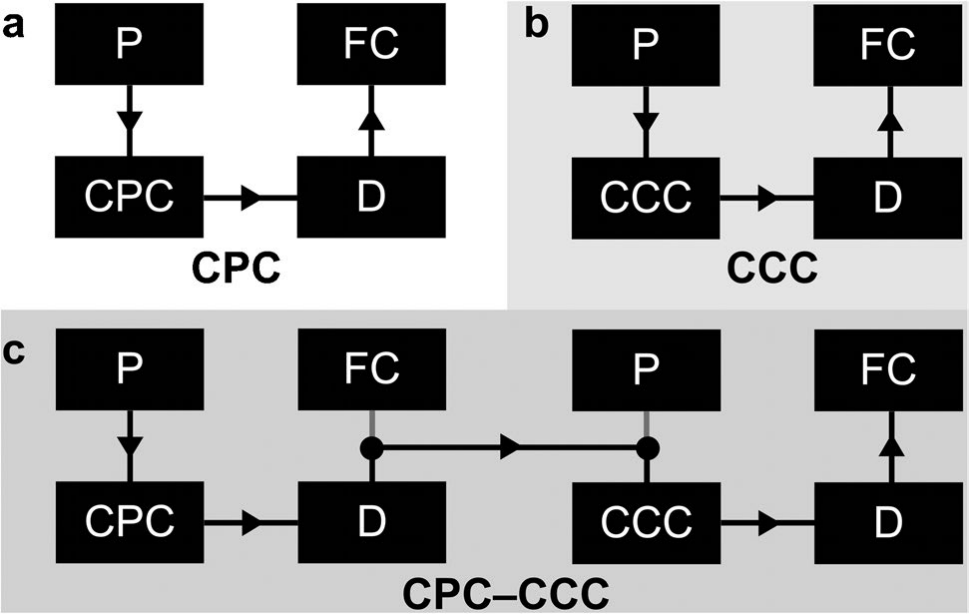
Schematic setup of the **a** CPC, **b** CCC, and **c** CPC-CCC separation (with P = pump, CPC = centrifugal partition chromatograph, CCC = countercurrent chromatograph, D = detector, FC = fraction collector) (black lines with drawn arrows show the direction of flow)

A CPC 250 PRO instrument (Gilson, Middleton, WI, USA) was used in combination with a flash 10 diode array detector (DAD; Ecom, Praha, Czech Republic) and a Gilson FC 204 fraction collector (Middleton, WI, USA) (Fig. 3a). The rotor of the CPC 250 PRO consists of 12 stainless steel disks with 20 engraved twin-cells each (240 cells in total) and has a total rotor volume of 250 mL. The instrument is specially designed for a larger sample quantity (up to 30 g) that is superior to those of a conventional CPC instrument of the same size, according to supplier information. The maximum values of achievable rotational speed and pressure drop are 3000 rpm (729 g) and 100 bar, respectively. The CPC rotor (filled with methanol after the last flush) was filled with stationary (upper) phase at a flow rate of 100 mL/min and a rotor speed of 500 rpm until its breakthrough. Then, the rotor speed was increased to 1600 rpm and the mobile phase was pumped with 2 mL/min through the system. Extruded stationary phase of 34 mL corresponded with a stationary phase retention (*S*_f_) of 86%. The sample (1 g dissolved in both 14.5 mL upper and lower phase) was injected via a 30-mL sample loop and the effluent was monitored at 210 nm. Between 70 and 630 mL, 80 × 7-mL fractions were collected. Two further separations with either 5-g (dissolved in both 12.5 mL upper and lower phase) or 10-g (dissolved in both 10 mL upper and lower phase) samples were performed in the same way with the exception of a higher mobile phase flow rate of 4 mL/min (*S*_f_ =  ~ 86%).

#### CCC system

A Quickprep MK8 instrument (AECS London, UK) combined with a flash 10 diode array detector (DAD; Ecom, Praha, Czech Republic) and a Gilson 203 B fraction collector (Middleton, WI, USA) (Fig. 3b) was used with the setup of Hammerschick et al. [23]. In brief, coil column system 2 + 3 (total volume 236 mL) was filled with stationary phase (ternary beta 50 pump, Ecom, Praha, Czech Republic) at a flow rate of 10 mL/min. The flow rate was reduced to 2 mL/min and rotation was set to 870 rpm. Mobile phase was introduced and the equilibrated system showed *S*_f_ = 86% (33 mL extruded stationary phase). One-gram sample (dissolved in 4.5 mL stationary and 4.5 mL mobile phase) was injected via a 10-mL sample loop. After injection, the CCC chromatogram was recorded at 210 nm and 80 × 7-mL fractions were collected in the range between 30 and 590 mL. A second run with 5-g sample, injected via a 30-mL sample loop, performed in the same way was accompanied with full loss of stationary phase (fractions were not collected).

#### Hyphenation of CPC with CCC (CPC-CCC)

Before the CPC-CCC run, the CPC system and the CCC system were individually equilibrated at a flow rate of 4 mL/min as shown in “CPC system” and “CCC system,” respectively. The CCC system (coil column system 2 + 3, 236 mL) showed *S*_f_ = 80% (47 mL extruded stationary phase) and the CPC system *S*_f_ = 86% (35 mL extruded stationary phase). After independent equilibration of both systems, online CPC-CCC was facilitated by connecting the effluent of the CPC system after the detector (“CPC system”) directly with the CCC inlet of mobile phase with PTFE tubing by means of two three-way valves (“CCC system”) (Fig. 3c). The dissolved sample (5 g in both 12.5 mL upper and lower phase) was injected via a 30-mL sample loop into the CPC system. The CPC-CCC run was monitored in both DADs (210 nm), and after 31.5 min, 124 × 7 mL fractions (126–994 mL) were directed to the fraction collector after the CCC. A second separation with 10-g sample (dissolved in both 10 mL upper and lower phases) was carried out under the same conditions.

#### Processing of CPC, CCC, and CPC-CCC fractions

Fractions of all runs were liberated from solvent by means of a rotational vacuum concentrator (10 mbar, 80 °C, 1500 rpm) (RVC 2–33 IR, Martin Christ, Osterode am Harz, Germany), and the masses of the residues were determined gravimetrically. Dry CCC, CPC, or CPC-CCC fractions were dissolved in 1 mL CE_az_, and aliquots of each fraction were silylated (“Sample preparation”) and measured by GC/MS. Elution profiles for the major ARs of all separations were generated according to Müller et al. [29]. Based on this protocol, purity profiles were created by plotting the respective percentage purities of the individual odd-chained AR peaks from the GC/MS chromatogram against the elution volume. Partially co-eluting minor ARs such as even-chained ARs, methylated ARs, and ARs with a keto group in the alkyl chain [27] were neither considered in plots nor in purity profiles. The resolution between the individual odd-chained AR peaks was calculated using the full width at half maximum of peaks (*ω*_0.5_) and the respective elution maxima (*V*_max_) (both graphically determined from the elution profiles) according to Eq. (1) [30]:

1 $$R= \frac{1.177 * [{\text{V}}_{\text{max}}\left(2\right) - {\text{V}}_{\text{max}}\left(1\right)]}{{\omega }_{0.5}\left(1\right) + {\omega }_{0.5}(2)}$$

### Gas chromatography with mass spectrometry (GC/MS)

Silylated aliquots of the transesterified and hydrogenated rye extract, as well as CPC, CCC and CPC-CCC fractions were analyzed on an HP G1800B GCD plus GC/MS system (Hewlett-Packard, Waldbronn, Germany). Aliquots of 1 μL were splitless injected with an HP 6890 series injector (Hewlett-Packard, Waldbronn, Germany). An HP-5MS column (30 m, 0.25 mm i.d., 0.25-μm film thickness, Agilent, Waldbronn, Germany) was supplied with a constant flow (1 mL/min) of the carrier gas helium. The GC oven program was 55 °C (1 min)–15 °C/min–250 °C–10 °C/min–320 °C (10 min) and temperatures of injector, transfer line, and ion source were maintained at 250 °C, 280 °C, and 160 °C, respectively. The GC/MS full scan range covered *m*/*z* 60–650 after a solvent delay of 7 min.

## Results and discussion

### CCS separation of saturated alkylresorcinols

Using the (more polar) lower phase as the mobile phase (head-to-tail mode in CCC, descending mode in CPC), ARs were eluted with increasing chain length and thus decreasing polarity. According to shake flask experiments with HEMWat − 7, *K*_U/L_ values (partition coefficients: ratio of analytes in upper (U) divided by lower (L) phase) of the analytes were 0.26 (AR15:0), 0.35 (AR17:0), 0.52 (AR19:0), 0.90 (AR21:0), 1.71 (AR23:0), and 2.35 (AR25:0) [23]. Further compounds in the hydrogenated and transesterified extract were saturated FAMEs (formed by transesterification and esterification) and sterols. These lipid classes are less polar and eluted later than the targeted ARs. Unsaturated ARs were saturated by hydrogenation. Accordingly, all interfering compounds that reduce mainly the purity of the saturated ARs were removed with the exception of low shares of even numbered and methyl-substituted ARs [27]. In addition, AR15:0 was partly co-eluting with ARs with a keto group in the alkyl chain.

### Single instrument technique

#### CCC vs. CPC separation of 1-g AR sample

**Fig. 4:**
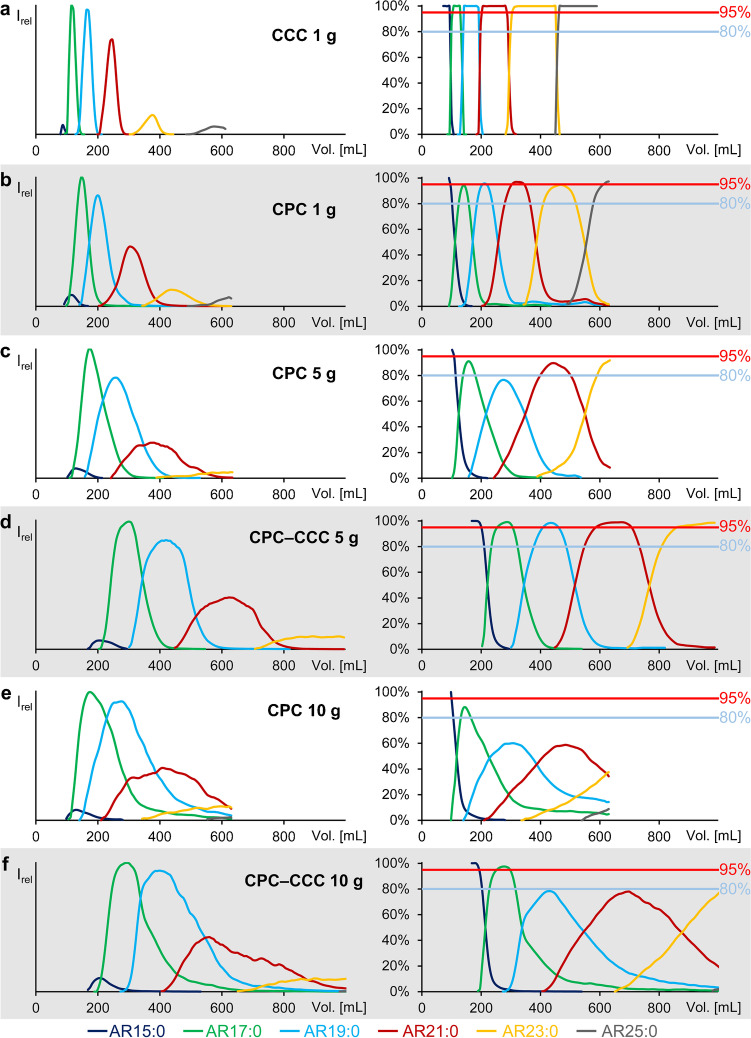
Elution profiles (left panel) and purity profiles (right panel) of the major alkylresorcinols of the **a** 1 g CCC, **b** 1 g CPC, **c** 5 g CPC, **d** 5 g CPC-CCC, **e** 10 g CPC, and **f** 10 g CPC-CCC separation

*K*_U/L_ values of ARs determined by shake flask experiments agreed well with those calculated from elution volumes in CCC (head-to-tail mode), i.e., *K* = 0.28 (AR15:0), 0.40 (AR17:0), 0.64 (AR19:0), 1.02 (AR21:0), 1.64 (AR23:0), and 2.60 (AR25:0) (Table 2). Accordingly, the individual saturated odd-chained ARs were (almost) baseline separated by CCC (Fig. 4a, left panel). The good peak resolution (*R*_(AR17:0/AR19:0)_ = 1.00, *R*_(AR19:0/AR21:0)_ = 1.29, *R*_(AR21:0/AR23:0)_ = 1.64, Table 3) not only enabled the isolation of the individual ARs in a very high purity (> 99.9%), but also in good yield (Fig. 4a, right panel, Table 2). Namely, CCC separation of 1 g methylated and hydrogenated rye extract by CCC provided 53 mg AR17:0, 78 mg AR19:0, 58 mg AR21:0, 29 mg AR23:0, and 12 mg AR25:0 with purities of > 95% (Table 2). In agreement with previous CCC runs [27], the CCC separation of 1-g sample was highly reproducible in terms of *K* values, yield, and purity.

**Table 2:** Masses (*m*) with a purity > 80% (and > 95%) (without consideration of the minor ARs) of the isolated saturated ARs of the CCC separation with 1 g, CPC separations with 1, 5, and 10 g, and the CPC-CCC separations with 5 and 10 g and their corresponding *K* values (calculated from the elution maximum) and *K*_U/L_ values from shake flask experiment according to Hammerschick et al. [23]

		AR15:0	AR17:0	AR19:0	AR21:0	AR23:0	AR25:0	Total *m* [mg]
Shake flask experiment	*K*_U/L_ value	0.26	0.35	0.52	0.90	1.71	2.35	-
CCC 1 g	*K* value	0.28	0.40	0.64	1.02	1.64	2.60	
	*m* purity > 80% (> 95%) [mg]	9.1 (9.1)	58.4 (52.8)	82.1 (77.6)	60.1 (57.9)	32.5 (29.1)	12.1 (11.7)	254 (238)
CPC 1 g	*K* value	0.38	0.52	0.77	1.27	1.88	-	
	*m* purity > 80% (> 95%) [mg]	8.4 (8.4)	57.5 (0)	61.8 (0)	56.1 (0)	30.9 (0)	11.0 (0)	226 (8.4)
CPC 5 g	*K* value	0.48	0.64	1.03	1.61	2.49	-	
	*m* purity > 80% (> 95%) [mg]	8.9 (8.9)	98.9 (0)	0 (0)	116 (0)	45.1 (0)	0 (0)	269 (8.9)
CPC-CCC 5 g	*K* value	0.33	0.52	0.85	1.34	2.08	-	
	*m* purity > 80% (> 95%) [mg]	32.3 (27.4)	305 (214)	410 (237)	320 (213)	123 (85.5)	-	1190 (780)
CPC 10 g	*K* value	0.49	0.65	1.07	1.75	2.56	-	
	*m* purity > 80% (> 95%) [mg]	19.4 (9.8)	133 (0)	0 (0)	0 (0)	0 (0)	0 (0)	152 (9.8)
CPC-CCC 10 g	*K* value	0.31	0.54	0.80	1.25	2.14	-	
	*m* purity > 80% (> 95%) [mg]	45.8 (38.1)	403 (205)	0 (0)	0 (0)	0 (0)	-	449 (243)

**Table 3:** Calculated resolutions R using peak width at half height between adjacent eluting ARs AR17:0, AR19:0, AR21:0, and AR23:0 for separation CCC 1 g, CPC 1 g, CPC 5 g, and CPC-CCC 5 g

Resolution R	CCC 1 g	CPC 1 g	CPC 5 g	CPC-CCC 5 g
AR17:0/AR19:0	1.00	0.55	0.44	0.57
AR19:0/AR21:0	1.29	0.83	0.44	0.59
AR21:0/AR23:0	1.64	0.79	Not determinable	Not determinable

Compared to CCC, CPC separation with 1-g sample (same mobile phase flow of 2 mL/min and *S*_f_ value (86%) as with CCC) resulted in slightly later elution times of ARs (Fig. 4b, left panel). Even though a larger rotor volume was used in CPC, which also slightly delayed the elution, *K* values (0.38 (AR15:0), 0.52 (AR17:0), 0.77 (AR19:0), 1.27 (AR21:0), and 1.88 (AR23:0)) calculated from the elution profile were higher than those in CCC (Table 2). More strikingly, however, the peaks in the CPC chromatogram were much broader than those in the CCC chromatogram (Fig. 4a,b, left panel). This intrinsically lower chromatographic efficiency of CPC was accompanied with worse peak resolutions (*R*_(AR17:0/AR19:0)_ = 0.55, *R*_(AR19:0/AR21:0)_ = 0.83, *R*_(AR21:0/AR23:0)_ = 0.79, Table 3). As a consequence, only small amounts of ARs could be obtained with purities > 95%, i.e., 27 mg AR21:0, 2 mg AR23:0, and 5 mg AR25:0 (Fig. 4b, right panel, Table 2). Even on the basis of purities of > 80%, the resulting total quantity of 226 mg ARs was lower than the amount of 238 mg ARs obtained by CCC with > 95% purity (Table 2). However, CPC can tolerate higher sample loads, which was evaluated in the next section.

#### CPC separation of 5- and 10-g AR sample (compared to 1 g)

Injection of 5- or 10-g samples into the equilibrated CPC system had no influence on the *S*_f_ value (no bleed). However, the higher sample loads increased the elution volumes of the ARs and partly caused peak tailing (Fig. 4c, e, left panel). Similarly, *K* values (calculated from the elution profile) were 0.48 (AR15:0), 0.64 (AR17:0), 1.03 (AR19:0), 1.61 (AR21:0), and 2.49 (AR23:0) for the 5 g injection (10 g injection gave very similar values) (Table 2). The reason for this could be the high mass of residual sample matrix (especially FAMEs [27]). According to Friesen and Pauli, increasing concentrations of solutes in the column due to a higher sample load will cause interactions in the form of attraction or repulsion with analytes which may delay their elution [31]. In the present case, attractions (solute–solute interactions are increasingly more relevant than solute–solvent interactions) were made responsible for a delayed elution. Accordingly, release of ARs may be delayed by attractive interactions between analytes and other sample compounds (especially FAMEs) and may result in peak tailing. Also, the peaks were broader (compared to 1-g sample) which in turn lowered the resolution between the individual ARs (5-g sample: *R*_(AR17:0/AR19:0)_ = 0.44, *R*_(AR19:0/AR21:0)_ = 0.44, Table 3). Overall, none of the 7-mL fractions featured ARs with purities > 95% (Fig. 4c, e, right panel) while the total amount of ARs with a purity > 80% in the 5 g injection (269 mg) was only slightly higher than with the 1-g sample (226 mg). Injection of 10-g sample into the CPC system resulted in an even lower total amount of 152 mg with > 80% purity (Table 2).

#### Conclusion of the single instrument techniques

According to expectations, the sample capacity in CPC was higher at the cost of the peak resolution while CCC showed a higher chromatographic efficiency, but was limited in the sample load. Specifically, injection of 5-g sample into the CCC (which was easily manageable in CPC) resulted in complete loss of stationary phase, because the maximum capacity was surpassed. In the following, we aimed to overcome the drawback of both methods by the hyphenation of both instruments.

### Separations with the hyphenated CPC-CCC system

For the hyphenation of both instruments, the flow rate was increased to 4 mL/min. Under this condition, the *S*_f_ value of the individually equilibrated CCC system was slightly lower (~ 80%) while the one of the CPC system was still at ~ 86%. After coupling of the instruments, injection of 5- and 10-g samples into the CPC system followed by full transfer into the CCC did not impair the stability. In agreement with our working assumption, the initial pre-separation in CPC strongly increased the sample capacity in CCC. This was partly due to the fact that FAMEs were not eluted from the CPC, which reduced the sample amount loaded into the CCC system. However, this point alone could not explain the stability after injection of 10-g sample whereby ~ 2.7 g total mass was transferred to the CCC system (based on fraction weights). Instead, due to the pre-separation during the CPC, separation with 10-g sample was generally < 45 mg/7 mL which corresponds with ~ 120 mg ARs/20 mL (Electronic Supplementary Material Table S1) which is the double volume of the injection loop with 1-g sample in CCC. Accordingly, the amount entering the CCC system was generally well below the capacity of 1 g lipids in the present instrument. Hence, CPC served both as injection tool and as an initial pre-separation step.

*K* values were approximately the same for both sample loads (5 g injection: 0.33 (AR15:0), 0.52 (AR17:0), 0.85 (AR19:0), 1.34 (AR21:0), 2.08 (AR23:0)). The resulting *K* values were between those of the CPC and CCC with 1 g each and those of the CPC separations with 5 g and 10 g, respectively (Table 2). In addition, the peak widths after CPC-CCC were virtually the same as after CPC alone. This indicated a higher chromatographic efficiency and sharper peaks refocused the individual ARs in the CCC instrument (Fig. 4c–f, left panel). In agreement with that, the resolution between the individual ARs improved by a factor of ~ 1.3 (5 g: *R*_(AR17:0/AR19:0)_ = 0.57, *R*_(AR19:0/AR21:0)_ = 0.59) (Table 3) which is close to the expected value of √2 due to the double column volume after CPC and CCC (250 + 236 mL; *R*_2_ = *R*_1_*√2 [32]). Most importantly, however, the higher initial sample load of 5- and 10-g samples strongly increased the amounts and partly provided good purities of ARs during the CPC-CCC run (Fig. 4c–f, right panel). Injections of 10-g sample allowed for the collection of very high amounts of ARs in the individual fractions (up to 50 mg). However, purities were only moderate due to peak tailing (Fig. 4e, f, right panel, Table 2). By contrast, injection of 5-g sample into the CPC-CCC setup provided an unmatched total amount of 780 mg ARs with a purity > 95% (214 mg AR17:0, 237 mg AR19:0, 213 mg AR21:0, 86 mg AR23:0) (Fig. 4d, right panel, Table 2). Fractions with ARs of > 80% purity increased the total amount to 1190 mg ARs (Table 2). This yield was much higher than what could be obtained with a single CCC run.

## Concluding remarks

Here we showed that CPC and CCC can easily be hyphenated just by the installation of switching valves and connection tube. As exemplified with the separation of ARs, the novel setup enabled higher sample loads and higher amounts of highly pure compounds compared with CCC alone. Injections of 5-g sample were manageable and provided higher amounts and purities of the analytes. The example of an injection of 10-g sample showed that limitations existed especially when peak tailing was increasing which reduced the purity in the individual fractions. Such problems could probably be reduced by cutting off heavily overlapping peaks in the first dimension. Namely, the three-way valve could direct the CPC effluent to waste (instead of directing it to the CCC) for a freely selectable period. This approach is similar to two-dimensional heart-cut separations as previously achieved with CCC [29, 33]. Similarly, uninteresting matrix compounds eluting late from the CPC must not be transferred into the CCC system (compare with FAMEs in the present study).

The novel CPC-CCC method will be particularly valuable for the enrichment and isolation of minor compounds or, more generally spoken, if the target compound is not very abundant in sample extracts. In this case, the beneficial effects of both hyphenated CCS methods will particularly come to fruition. Namely, injection into the CPC manages high sample loads (and pre-separates the “matrix”) while the subsequent CCC separation will provide the best possible purity. Another application of this approach would be to use two different solvent systems in the two instruments. However, care would have to be taken that the solvent system in the CCC remains stable after the transfer of a certain elution range from the CPC effluent directly to the CCC and that no flooding of the stationary phase occurs. Hence, facilitation of the direct CPC-CCC coupling with the described advantages and perspectives could be interesting for different users with CPC and CCC systems at hand. The hyphenated CPC-CCC technique is expected to work not only for alkylresorcinols but for all classes of molecules or biomolecules that can be separated with a suitable biphasic solvent system, which should be stable in both CPC and CCC.

## Supplementary Information

Below is the link to the electronic supplementary material.

Supplementary file1 (DOCX 22 KB)

## Abbreviations

AR: Alkylresorcinol
CCC: Countercurrent chromatography
CCS: Countercurrent separation
CPC: Centrifugal partition chromatography
CE_az_: Azeotropic mixture of cyclohexane/ethyl acetate
FAME: Fatty acid methyl ester
ISTD: Internal standard
ME: Methyl ester
*S*_f_: Retention of the stationary phase
