# Supplementary material for: Online hyphenation of centrifugal partition chromatography with countercurrent chromatography (CPC-CCC) and its application to the separation of saturated alkylresorcinols
Source: Anal Bioanal Chem. 2022 May 31;414(17):5043–51. doi: 10.1007/s00216-022-04136-x (PMC9234026; doi:10.1007/s00216-022-04136-x)
Supplement: Supplementary file 1 — Supplementary file1 (DOCX 22 KB) [file 216_2022_4136_MOESM1_ESM.docx]

**Online hyphenation of centrifugal partition chromatography with countercurrent chromatography (CPC–CCC) and its application to the separation of saturated alkylresorcinols**

Tim Hammerschick^1^ and Walter Vetter ^1^*

^1^ University of Hohenheim, Institute of Food Chemistry, Department of Food Chemistry (170b), D-70599 Stuttgart, Germany

* Corresponding author

Walter Vetter

Email: [walter.vetter@uni-hohenheim.de](mailto:walter.vetter@uni-hohenheim.de)

(ORCID: 0000-0002-5592-4265)

**Table S1** Mass of fractions of CPC separation with 10 g sample (corresponds to the mass transferred to CCC in CPC–CCC separation after CPC)

| Fraction | Elution volume [mL] | Mass per fraction [mg] |
| --- | --- | --- |
| 1 | 77 | 10.5 |
| 2 | 84 | 10.7 |
| 3 | 91 | 9.7 |
| 4 | 98 | 9.8 |
| 5 | 105 | 9.6 |
| 6 | 112 | 10.4 |
| 7 | 119 | 12.4 |
| 8 | 126 | 15.3 |
| 9 | 133 | 19.9 |
| 10 | 140 | 24.0 |
| 11 | 147 | 27.7 |
| 12 | 154 | 29.6 |
| 13 | 161 | 31.4 |
| 14 | 168 | 32.8 |
| 15 | 175 | 34.4 |
| 16 | 182 | 35.4 |
| 17 | 189 | 37.1 |
| 18 | 196 | 37.9 |
| 19 | 203 | 38.9 |
| 20 | 210 | 40.1 |
| 21 | 217 | 40.7 |
| 22 | 224 | 41.4 |
| 23 | 231 | 40.6 |
| 24 | 238 | 40.0 |
| 25 | 245 | 39.6 |
| 26 | 252 | 38.8 |
| 27 | 259 | 38.6 |
| 28 | 266 | 37.9 |
| 29 | 273 | 37.2 |
| 30 | 280 | 35.7 |
| 31 | 287 | 35.8 |
| 32 | 294 | 34.8 |
| 33 | 301 | 35.0 |
| 34 | 308 | 34.1 |
| 35 | 315 | 33.0 |
| 36 | 322 | 32.0 |
| 37 | 329 | 32.6 |
| 38 | 336 | 29.8 |
| 39 | 343 | 29.2 |
| 40 | 350 | 27.6 |
| 41 | 357 | 26.6 |
| 42 | 364 | 25.5 |
| 43 | 371 | 24.9 |
| 44 | 378 | 24.3 |
| 45 | 385 | 23.3 |
| 46 | 392 | 22.2 |
| 47 | 399 | 22.0 |
| 48 | 406 | 21.8 |
| 49 | 413 | 21.2 |
| 50 | 420 | 20.4 |
| 51 | 427 | 19.4 |
| 52 | 434 | 18.6 |
| 53 | 441 | 18.9 |
| 54 | 448 | 18.3 |
| 55 | 455 | 18.2 |
| 56 | 462 | 17.5 |
| 57 | 469 | 17.2 |
| 58 | 476 | 16.8 |
| 59 | 483 | 16.5 |
| 60 | 490 | 15.4 |
| 61 | 497 | 14.8 |
| 62 | 504 | 14.5 |
| 63 | 511 | 14.4 |
| 64 | 518 | 13.0 |
| 65 | 525 | 14.6 |
| 66 | 532 | 12.5 |
| 67 | 539 | 11.9 |
| 68 | 546 | 11.7 |
| 69 | 553 | 11.0 |
| 70 | 560 | 10.8 |
| 71 | 567 | 10.5 |
| 72 | 574 | 10.7 |
| 73 | 581 | 11.6 |
| 74 | 588 | 12.5 |
| 75 | 595 | 9.7 |
| 76 | 602 | 9.6 |
| 77 | 609 | 9.0 |
| 78 | 616 | 8.7 |
| 79 | 623 | 8.5 |
| 80 | 630 | 8.3 |
